# Pediatric traumatic brain injury: Language outcomes and their relationship to the arcuate fasciculus

**DOI:** 10.1016/j.bandl.2013.05.003

**Published:** 2013-12

**Authors:** Frédérique J. Liégeois, Kate Mahony, Alan Connelly, Lauren Pigdon, Jacques-Donald Tournier, Angela T. Morgan

**Affiliations:** a Developmental Cognitive Neuroscience Unit, University College London, Institute of Child Health, London, United Kingdom; b Great Ormond Street Hospital for Children, London, United Kingdom; c Brain Research Institute, Florey Neuroscience Institutes, Melbourne, Australia; d Murdoch Children’s Research Institute, Melbourne, Australia; e Department of Paediatrics, University of Melbourne, Australia

**Keywords:** Pediatric brain injury, Expressive language, Dysarthria, Tractography, Arcuate fasciculus.

## Abstract

Pediatric traumatic brain injury (TBI) may result in long-lasting language impairments alongside dysarthria, a motor-speech disorder. Whether this co-morbidity is due to the functional links between speech and language networks, or to widespread damage affecting both motor and language tracts, remains unknown.

Here we investigated language function and diffusion metrics (using diffusion-weighted tractography) within the arcuate fasciculus, the uncinate fasciculus, and the corpus callosum in 32 young people after TBI (approximately half with dysarthria) and age-matched healthy controls (*n* = 17). Only participants with dysarthria showed impairments in language, affecting sentence formulation and semantic association. In the whole TBI group, sentence formulation was best predicted by combined corpus callosum and left arcuate volumes, suggesting this “dual blow” seriously reduces the potential for functional reorganisation. Word comprehension was predicted by fractional anisotropy in the right arcuate. The co-morbidity between dysarthria and language deficits therefore seems to be the consequence of multiple tract damage.

## Highlights

• Diffusion weighted MRI can assist in the prognosis of neuropsychological deficits.
• We explored white matter changes and language outcome in people with brain injury.
• Sentence generation impairments were found in dysarthric participants.
• Impairments were linked to reduced corpus callosum and left arcuate fasciculus size.
• This dual blow seriously reduces the potential for language reorganisation.

## Graphical abstract

**Figure:**
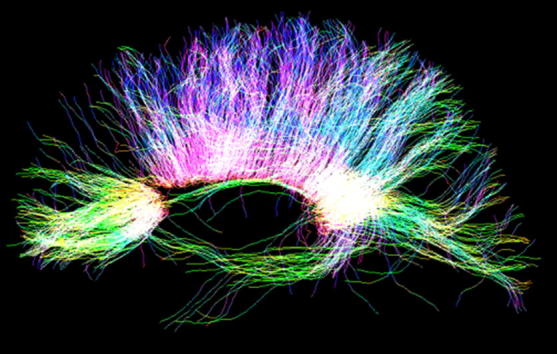

## 1 Introduction

Traumatic brain injury (TBI) in childhood is a serious public health problem worldwide due to its high prevalence (e.g., 91 per 100,000 in Australia; Berry, Jamieson, & Harrison, 2010) and morbidity rates. Sustaining a TBI in childhood has been found to cause persistent, diverse and complex neuropsychological impairments across cognitive domains (Anderson, Godfrey, Rosenfeld, & Catroppa, 2012; Anderson, Morse, Catroppa, Haritou, & Rosenfeld, 2004; Babikian & Asarnow, 2009; Crowe, Catroppa, Babl, & Anderson, 2012; Taylor, 2004), including chronic language impairments (Anderson et al., 2004; Jordan & Murdoch, 1990), especially if the injury is severe. Given the varied and often widespread nature of brain white matter damage after TBI (Smith, Meaney, & Shull, 2003), it is not surprising that the neural correlates of persistent language impairments remain elusive.

Childhood acquired language disorders may be characterised by deficits in any or all linguistic domains of vocabulary, pragmatics, syntax, morphology or semantics (Feldman & Messick, 2008). ‘Higher-order’ language is particularly impaired in pediatric and adult TBI, including discourse skills (Lê, Coelho, Mozeiko, Krueger, & Grafman, 2012; Marini et al., 2011) and understanding of irony or non-literal concepts (Angeleri et al., 2008; Dennis, Purvis, Barnes, Wilkinson, & Winner, 2001). However, factors such as severity of TBI and age at injury are reported to influence outcome, and younger children with severe TBI may present with impairments in both lower- (e.g., semantic, syntactic) and higher-order levels of language (Anderson et al., 2004; see Sullivan & Riccio, 2010 for a review).

Dysarthria, a motor-speech disorder, is also a common occurrence after TBI and may affect the intelligibility of the speaker (Morgan, Mageandran, & Mei, 2010). It can result from a combination of respiratory, phonatory, articulatory, and/or resonatory impairments (Cahill, Murdoch, & Theodoros, 2002). Although distinct functions, language deficits and dysarthria can both result following pediatric TBI, with reported co-morbidity rates being as high as 55% (Morgan et al., 2010). It is well established that both long-range distance and U-shaped white matter connections exist between traditional language regions such as Broca’s area and primary motor regions (Catani et al., 2011). It is therefore possible that damage to speech motor regions may have a direct impact on language production regions. Whether the co-morbidity between dysarthria and language impairments is due to the functional links between speech and language networks, or to widespread injury affecting both motor and language tracts, remains unknown.

TBI can result in heterogeneous neural injuries, causing both global and focal lesions (see Gaetz, 2004 for a review), with diffuse axonal injury being most commonly observed. Diffusion-weighted magnetic resonance imaging (DWI) methods are optimal for detecting changes in white matter that are assumed to reflect microstructural changes (Beaulieu, 2002; Marquez de la Plata et al., 2011; Sundgren et al., 2004) such as those seen in TBI. Tractography is one specific DWI-derived technique that permits the tracing and reconstruction of white matter tracts in vivo. This approach is proving to be clinically useful (Ciccarelli, Catani, Johansen-Berg, Clark, & Thompson, 2008), for example, in identifying neural biomarkers and improving prognostic accuracy of long-term neuropsychological functioning after TBI in adults (e.g., Huisman et al., 2004; Shenton et al., 2012; Wang et al., 2011) and in children (Johnson et al., 2011; Oni et al., 2010; Tasker, Westland, White, & Williams, 2010). Specifically, fractional anisotropy (FA) has been used to infer the structural “integrity” of the tract – which animal models have suggested may be related to demyelination and axonal degeneration (Budde, Janes, Gold, Turtzo, & Frank, 2011). FA has been shown to increase with age in most white matter tracts during normal development (Trivedi et al., 2009), including those involved in language function (Lebel & Beaulieu, 2009) and the presence of TBI is likely to affect this developmental trajectory.

Language functions rely on an extensive network of short- and long-range connections, both ventral and dorsal to the sylvian fissure (e.g., Glasser & Rilling, 2008; Saur et al., 2008). The arcuate fasciculus (AF) is part of the “dorsal pathway” and corresponds to the long segment of the superior longitudinal fasciculus that connects Broca’s and Wernicke’s areas (Catani, Jones, & Ffytche, 2005). The AF is considered a major language tract, and studies of healthy adults have demonstrated a relationship between its symmetry and performance on a word list recall task (Catani et al., 2007). AF abnormalities have been documented in pediatric populations that exhibit language impairments of varying severity, as part of a more globally affected profile including cognitive involvement, such as Angelman syndrome (Wilson et al., 2011), bilateral perisylvian syndrome (Saporta, Kumar, Govindan, Sundaram, & Chugani, 2011), autism (Fletcher et al., 2010) and in developmental populations with a primary or specific language impairment (SLI children, who have language deficits despite adequate development, typical intelligence and language learning opportunity, Verhoeven et al., 2011). Whether persisting language impairments after childhood TBI can be predicted by examining the diffusion properties of the AF remains to be investigated.

In contrast to the arcuate fasciculus, the uncinate fasciculus is part of the “ventral” language pathway (Parker et al., 2005) and connects the anterior temporal lobe to the orbitofrontal cortex. This tract has recently been the focus of renewed interest in the language literature (Axer, Klingner, & Prescher, in press; Weiller, Bormann, Saur, Musso, & Rijntjes, 2011), and together with the extreme capsule system, is an integral part of the semantic network (Friederici, 2012; Saur et al., 2008; Vigneau et al., 2006). Because it runs through the extreme capsule and goes through the temporal stem, the uncinate fasciculus is vulnerable to TBI (Bigler et al., 2010). Changes in the diffusion properties of this tract may therefore explain a significant proportion of language outcome, especially semantic deficits, in young people who have suffered a TBI in childhood.

Finally, we have recently reported that corpus callosum integrity (especially the splenium) was the main predictor of language impairments in adolescents born very preterm (Northam et al., 2012). Another study in a small group of children with idiopathic language impairment reported a significant FA reduction in the genu of the corpus callosum relative to healthy controls (Kim et al., 2006). These results point towards a crucial role of inter-hemispheric connections (between the temporal lobes in Northam et al. (2012)) in language development and processing. The role of inter-hemispheric connections may be particularly important during language development where a shift from inter- to intra-hemispheric connectivity has been suggested between childhood and adulthood (Friederici, Brauer, & Lohmann, 2011). Given that the corpus callosum is particularly vulnerable to shearing and stretching during TBI (both acutely and chronically, Ewing-Cobbs et al., 2008; Wang et al., 2011; Wu et al., 2011), we also investigated whether damage to this tract could explain some of the language variance in our sample.

Here we sought to examine the relationship between white matter integrity and persistent language impairment after pediatric TBI, with and without concomitant dysarthria. First we aimed to investigate whether poor language outcome long-term following a TBI in childhood can be predicted by changes in tractography-derived properties that are measured within the arcuate fasciculus, the uncinate fasciculus, and the corpus callosum. We hypothesised that language impairments would be found in children with TBI regardless of dysarthria, with variance in language ability being partially explained by cumulative effects of tract abnormalities. Secondly, we examined which MRI measure was the best predictor of language outcome after childhood TBI. Finally, we explored whether age at injury and injury severity affect outcomes in our sample (Anderson, Brown, Newitt, & Hoile, 2011).

## 2 Materials and methods

### 2.1 Participants

Data were collected as part of a wider study investigating neural correlates of speech and language outcomes after pediatric TBI. Forty-nine young people (22 males) who completed both the neuropsychological assessment and MRI scanning sessions participated in this study. Thirty-two (14 males) had sustained a traumatic brain injury (TBI) in childhood (3–16 years, see Table 1, and also Table 1 in Morgan, Masterton, Pigdon, Connelly, and Liégeois (2013), for individual data) at least 1 year earlier and were recruited from the medical records of the Royal Children’s Hospital Melbourne (RCH), in Australia. A subset of this sample had been diagnosed with dysarthria as a result of their traumatic brain injury (TBI+ group), while the remainder did not (TBI− group, see Table 1). Breakdown of injury severity ranged from mild to moderate–severe using the Mayo injury severity scale (Malec et al., 2007), sustained in various accidents (see Table 1, and also Table 1 in Morgan et al. (2013), for individual injury characteristics). We also collected GCS scores for each participant, coded as Mild (scores >12), Moderate (scores were between 9 and 12), or Severe (scores <9). The GCS is limited in being a single indicator of injury severity, where scores may be influenced by factors such as whether or not early sedation was utilised and the timing of when the score was taken post-injury (see Malec et al., 2007 for further review and discussion). GCS scores were recoded categorically as severe (3), moderate (2) or mild (1) for statistical analyses.

A typically developing group (‘Control’ group) was also recruited from the community. Each control participant was individually age- and sex-matched to two TBI participants, one of whom had dysarthria and one who did not. The majority of participants were right handed (14/17 in the TBI+ and TD group, 13/15 in the TBI− group).

Ethics approval was obtained from the Royal Children’s Hospital Human Research Ethics Committee, approval number 27083. All participants gave informed consent and assent (if aged under 18). All guardians of participants gave informed consent.

### 2.2 Neuropsychological assessment

All participants underwent neuropsychological assessments for language and intellectual functioning, completed as part of a wider speech motor assessment protocol at the Brain Research Institute (BRI), Melbourne, for 2 h. They completed four core subtests of the Clinical Evaluation of Language Fundamentals (4th edition, Australian) as a measure of overall language ability (CELF-IV: Semel, Wiig, & Secord, 2006). Only the three subtests that were common to both age groups were used in the statistical analyses here.

The “Recalling Sentences” subtest assesses the ability to recall and reproduce exact sentence structure with increasing lengths and levels of syntactic complexity. “Formulated Sentences” assesses the ability to formulate complete, semantically and grammatically correct spoken sentences using given words or short phrases that vary in frequency of use and complexity, with a contextual prompt in the form of picture stimuli.

“Word Classes” assesses the ability to comprehend relationships in the meaning of words and to form word associations. The ‘total’ word classes score was used here, which combines scores from both the receptive (i.e., being able to list which 2 out of a possible list of 4 words are related, e.g., pillow, door, blanket, lamp) and expressive (i.e., being able to explain how the words are related, e.g., They are both things we sleep on) components of the subtest.

The two-subtest version of the Wechsler Abbreviated Scale of Intelligence (WASI, Wechsler, 2011), consisting of Matrix Reasoning and Vocabulary subtests, was used to measure overall intellectual ability.

### 2.3 MRI acquisition

All participants were scanned with a 3 T magnetic resonance imaging (MRI) scanner (Siemens Trio Tim 3 T MRI scanner, Erlangen, Germany) at the Brain Research Institute, Melbourne, for a total time of 37 min. The T1- and T2- (using dual echo) weighted structural images, high resolution whole brain 3D structural images, echo planar imaging (EPI) data, diffusion weighted images (DWI) and fMRI data were acquired during this session as part of the wider study (Liégeois, Tournier, Pigdon, Connelly, & Morgan, 2013; Morgan et al., 2013). The DWI images were obtained using gradients at 64 evenly spaced directions at a *b*-value of 3000 s/mm^2^ (TE/TR = 110/8300 ms, FOV = 240 × 240 mm, matrix size = 96 × 96, slice thickness = 2.5 mm (isotropic voxel size = 2.5 × 2.5 × 2.5 mm), 60 contiguous axial slices (as in Liégeois et al. (2013)).

### 2.4 DWI processing and analysis

Analysis of the DWI images was performed in native space using the MRtrix software package (Tournier, Calamante, & Connelly, 2012) based on the probabilistic streamlines method, combined with the constrained spherical deconvolution (CSD) technique to model multiple fibre-orientations. Constrained spherical deconvolution is particularly suitable for the tracking of the arcuate and uncinate fasciculi because of its superior ability to reliably track fibres in areas containing crossing fibres. The probabilistic algorithm employed in MRtrix works by taking small steps (0.2 mm by default) along drawn directly from the CSD-derived fibre orientation distribution (FOD) using rejection sampling. Full details are provided in Tournier et al. (2012).

**Fig. 1:**
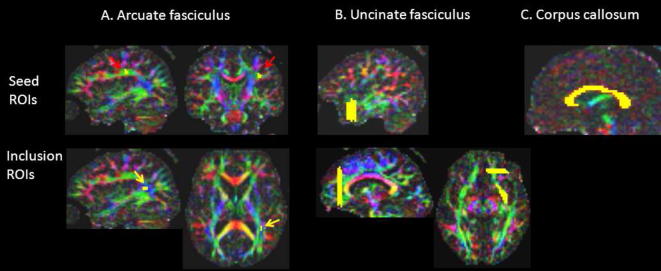
Location of seed and inclusion Regions of Interest (ROIs) for the arcuate fasciculus, uncinate fasciculus and corpus callosum. Colours on each eigenvector map represent the main direction of white matter tracts (red: left–right, blue: cranio-caudal, green: antero-posterior). See text for anatomical landmarks used.

Tractography was conducted manually and in native space according to a set protocol in each hemisphere for each subject (see Fig. 1 for illustration), with all ROIs delineated within MRtrix using a one-voxel paintbrush. Briefly, for the arcuate fasciculus, the seed region was a three-slice thick volume delineated on consecutive coronal slices, at the level of the classical arcuate fasciculus “bottleneck” (as in Galantucci et al., 2011). This region is coded green on the eigenvector map – indicating an antero-posterior orientation (Fig. 1A). The inclusion region was delineated across three consecutive slices in the axial plane in the superior temporal gyrus, coded blue on the eigenvector maps (dorsal–ventral direction; Fig. 1A).

The uncinate fasciculus was tracked using three large ROIs, one in the extreme capsule (delineated across three consecutive axial slices), one in the anterior temporal lobe (seed region, encompassing the whole antero-posterior white matter across 5 coronal slices). The third inclusion ROI was a 3-slice thick cross section of the anterior frontal lobe, immediately anterior to the cingulate bundle (see Fig. 1B for illustration). The corpus callosum was tracked using a single seed ROI from a manually delineated entire sagittal cross section (three slices thick, red on the eigenvector map, see Fig. 1C). Tailoring our ROI sizes to encompass the distinguishing anatomical features of the three tracts examined enabled immediate and reliable tract identification, without the need for post hoc exclusion ROIs.

The maximum number of streamlines generated was limited to 100,000 and the maximum number of streamlines selected was set at 1000. To avoid the inclusion of spurious thin tracks in the calculation of arcuate and uncinate volumes, the generated tracks were thresholded to include voxels including a minimum of 10 streamlines. All voxels with 10 or more streamlines passing through were included in the volume calculation, without weighting.

### 2.5 Tractography-derived measures

The above-mentioned thresholded tracks were converted into binary masks from which volumes (voxel count) and mean FA were extracted within MRtrix. Global white matter volumes had already been extracted using the Easyvolume toolbox implemented in SPM8.

For 12 participants (60 tracks), tractography was performed again by the same experimenter. Intra-rater reliability was excellent for both mean FA (Cronbach’s alpha range: 0.92 for the left uncinate to 0.99 for the left arcuate), and track volume (Cronbach’s alpha range: 0.90 for the right uncinate to 0.99 for the corpus callosum). For the right arcuate volume reliability was acceptable (alpha = 0.76).

### 2.6 Statistical analysis

One-way analyses of variance (ANOVA) investigated the main effects of Group on IQ, language, and tractography-derived measures. We used multivariate analyses to examine group differences across the three CELF-IV subtests, with IQ as a covariate (MANCOVA). To examine effects of track reduction beyond global white matter, analyses of co-variance (ANCOVA) were run with global white matter included as a covariate when examining volume differences. The impact of age at injury on language outcome and diffusion metrics was investigated using correlations. First, we ran simple correlations between tractography measures and outcome measures to isolate meaningful predictors. In a second step, we ran regression analyses.

Linear regressions were used to investigate the best tractography-derived predictors of language performance in the two TBI groups combined. The backward method was used to reduce the risk of Type II error. All predictors were entered in the model as a first step. Any predictor that did not make a significant contribution to the model was then removed, and the model was re-estimated. If this significantly weakened the model, then the predictor variable was re-entered – otherwise, it was deleted. This procedure was then repeated until only useful predictor variables remained in the model. The backward method was chosen as it is less likely to miss a predictor of outcome than the forward method.

## 3 Results

### 3.1 Descriptive measures

The three groups were adequately matched for age (*F*(2, 46) = 0.18, *p* = 0.84). Similarly, there were no differences between the two TBI groups on any injury related measure (*p* > 0.25 in all cases). In numerous cases, cortical injury was present in the acute stage (Supplementary Table 1). All groups typically had IQ and language scores within the normal range (Table 2), with the TBI+ group falling within the low end of this range. There was a strong relationship between language (CELF-IV score) and intellectual functioning in this TBI sample (*r*(30) = 0.76, *p* < 0.001). In the TBI groups, only five individuals (16%) would be classified clinically as language impaired, as defined by a CELF-IV standardised score below 85 (i.e., 1 SD below the mean), with four of those participants being from the TBI+ group. In the TBI+ group, six participants (35%) had standard scores one SD below the norm (7 or less) on the Formulated Sentences subtest, four on the Recalling Sentences subtest, and two on the Word Classes subtest.

### 3.2 Group differences in language ability

A one way ANCOVA with IQ as a covariate revealed a main effect of group (*F* = 3.50, *p* = 0.04; partial Eta squared = 0.14), with post hoc comparisons (Bonferroni correction) indicating that the group with dysarthria performed statistically significantly worse on the CELF-IV than the healthy control group (*p* = 0.04).

**Fig. 2:**
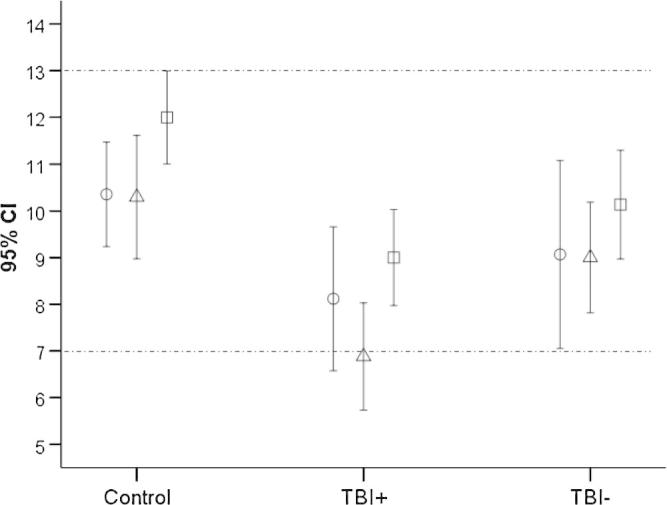
Mean language subtest standardised scores for the CELF-IV across the three groups. Circles, Recalling Sentences; triangles, Formulated Sentences; squares, Word Classes. Normal mean ±1 SD is indicated between the dotted lines.

MANCOVA (IQ as a covariate) on the three subtests indicated a statistically significant difference between the three groups on the combined dependent variable, *F*(3, 46) = 2.52, *p* = 0.03; partial Eta squared = 0.15). Two subtests showed significant group differences, namely Formulated Sentences (*F* = 4.32, *p* = 0.02; partial Eta Squared = 0.16) and Word Classes (*F* = 4.96, *p* = 0.01; partial Eta Squared = 0.18; see Fig. 2, and Supplementary Fig. 1 for boxplots and Supplementary Fig. 2 for ANCOVA plots). Post hoc tests revealed that the TBI+ group scored significantly lower than the healthy control group on both measures (*p* < 0.02 in both cases). When IQ was not used as a covariate, these effects were of greater magnitude (partial Eta squared >0.28), and additionally, the TBI+ group was found to perform lower than the TBI− group on Formulated Sentences (*p* = 0.045).

Altogether, the behavioural data suggest that young people who have sustained a TBI in childhood and have dysarthria are at a relative increased risk of language impairment, especially in the domains of sentence generation and word comprehension. These deficits are present beyond the level predicted by variance in their intellectual function.

### 3.3 Group differences in tractography-derived measures

**Fig. 3:**
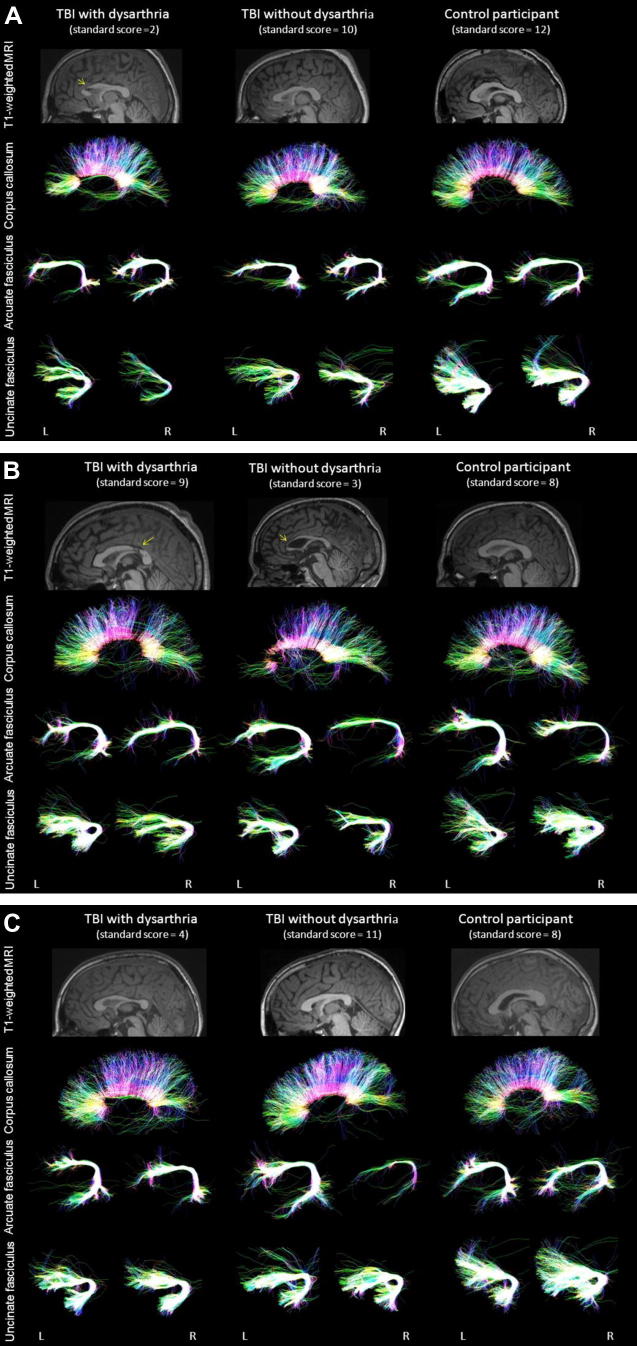
Illustrative examples of tractography reconstruction of the corpus callosum, arcuate fasciculus, and uncinate fasciculus in three age matched participants (A, 17 year-old males; B, 21-year-old males; C, 12 year-old males). Standard scores on the Formulated Sentences subtest are indicated in brackets for each participant. The yellow arrow indicates visible lesion of the corpus callosum. Sagittal projections are colour-coded for dominant direction of fibres (red: left–right, blue: cranio-caudal, green: antero-posterior). L, left hemisphere; R, right hemisphere. Note the effect of TBI on track macrostructure.

Tractography was successful for all participants. In several instances however, no voxel survived thresholding, and therefore a volume of 0 was attributed and the corresponding FA value was treated as missing value (one left uncinate, five right uncinate, and seven right arcuate fasciculi) (see Fig. 3 for illustrative examples).

**Fig. 4:**
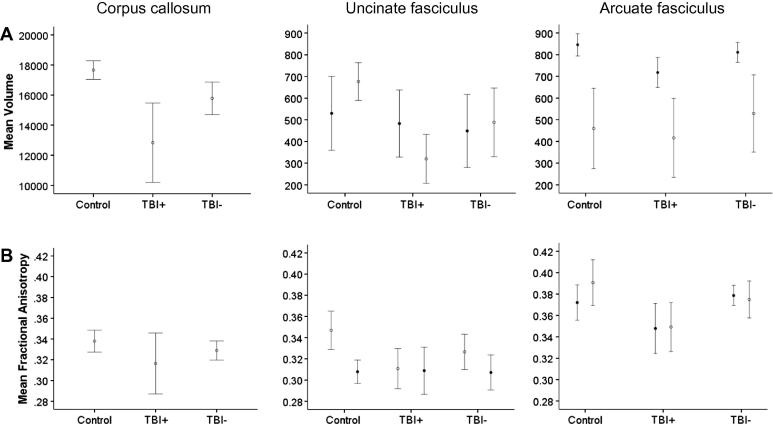
Mean track volume (A) and FA (B) for each participant group. Filled circles, left hemisphere; empty circles, right hemisphere. Error bars indicate 95% confidence intervals. See Table 3 for statistical comparisons.

In addition to differences in corpus callosum, uncinate and arcuate measures relative to the healthy control group (Table 3), the TBI group with dysarthria had reduced FA in the left arcuate fasciculus and reduced volume in both the corpus callosum and left arcuate relative to their peers without dysarthria (Table 3 and Fig. 4; see Supplementary Fig. 3 for boxplots).

### 3.4 Tractography-derived predictors of language outcome in the TBI group

#### 3.4.1 Formulating sentences

Linear correlations indicated that Formulated Sentences subtest scores were positively correlated with FA of the left arcuate fasciculus (*r*(30) = 0.38, *p* = 0.03), volume of the left arcuate (*r*(30) = 0.42, *p* = 0.018), and volume of corpus callosum (*r*(30) = 0.49, *p* = 0.005; see Supplementary Fig. 4A).

Regressions with the above measures as predictors revealed that the model that explained the highest proportion of variance included volume metrics of both the corpus callosum and left arcuate fasciculus (*F* = 6.48, *p* = 0.005; adjusted *R*^2^ = 0.26). It is noteworthy that volume of the corpus callosum alone and volume of the left arcuate fasciculus alone were significant and unique predictors of scores on this subtest as well (*F* = 9.38; *p* = 0.005; *β* = 0.49, adjusted *R*^2^ = 0.21 for corpus callosum; *F* = 6.32, *p* = 0.02; *β* = 0.42, adjusted *R*^2^ = 0.15 for arcuate fasciculus).

#### 3.4.2 Word Classes

Linear correlations indicated that the Word Classes subtest scores were positively correlated with FA of the right arcuate fasciculus (*r*(25) = 0.41, *p* = 0.03) and volume of the left uncinate (*r*(30) = 0.44, *p* = 0.01; see Supplementary Fig. 4B). Regressions with the above measures as predictors revealed that a model with both predictors was significant (*F* = 3.95, *p* = 0.03; adjusted *R*^2^ = 0.19), as was a model with FA of the right arcuate fasciculus alone (*F* = 5.00, *p* = 0.03; adjusted *R*^2^ = 0.13, *β* = 0.41).

#### 3.4.3 Recalling Sentences

Linear correlations indicated that the Recalling Sentences subtest scores were only positively correlated with FA of the right arcuate fasciculus (*r*(25) = 0.44, *p* = 0.02; see Supplementary Fig. 4C). Regression indicated that the model with this predictor was significant (*F* = 5.83, *p* = 0.02; adjusted *R*^2^ = 0.16, *β* = 0.44).

### 3.5 Effect of injury characteristics on language outcome

Severity of injury (using Glasgow-Coma Scale score) and age at injury did not correlate with any language measure, whether TBI participants were examined as a single group or as two groups separately. Similarly, global white matter volume did not correlate with any language measure.

### 3.6 Effect of injury characteristics on tractography-derived measures

Severity of injury assessed using the Glasgow-Coma scale classification was negatively correlated with volume of the right uncinate fasciculus only (*r*(30) = −0.47, *p* = 0.007). Age at injury (but not age) was positively correlated with volume of the left arcuate fasciculus (*r*(30) = 0.38, *p* = 0.03).

### 3.7 Correlations between language measures

Although the Formulated Sentences subtest was the most impaired in the dysarthric sample studied here, scores on this subtest were highly correlated with both the Word Classes (*r*(30) = 0.44, *p* = 0.01) and Recalling Sentences (*r*(30) = 0.67, *p* < 0.001) scores.

### 3.8 Relationships between language and motor track metrics

Tractography derived data were compared to those of a previous study by our group examining motor tracts in this cohort (Liégeois et al., 2013) in exploratory analyses. In that study, we used tractography to map two portions of the corticobulbar tract, namely (i) a dorsal pathway originating from the lip/jaw somatotopic representation, and (ii) a more ventral pathway originating from the tongue representation (see Liégeois et al., 2013, for details). Volumes and mean FA were extracted, as reported here. There was a positive correlation between volume of the left arcuate and volume of (i) both dorsal corticobulbar tracks, and (ii) right ventral corticobulbar tracks.

## 4 Discussion

Here we examined whether co-morbid speech motor and language deficits after TBI are due to the functional links between speech and language networks, or to widespread damage affecting both motor and language tracts. Specifically, we studied the long-term impact of TBI in childhood on language related tracts and the corpus callosum, in people with and without dysarthria. The group with dysarthria performed significantly more poorly relative to the other groups, mainly in the area of sentence production and word comprehension. Sentence production was related to macroscopic changes within the left arcuate fasciculus and corpus callosum, beyond global white matter reduction. Performance on word comprehension was related to changes in the right arcuate fasciculus.

### 4.1 Language outcomes

Our results have shown that language ability is compromised in the years after pediatric TBI, in comparison to normal peers, and that this deficiency can be observed beyond IQ differences. This risk to language performance was relatively higher in those with dysarthria, who scored on average eight points (or just over half a standard deviation) below their non-dysarthric peers. Most TBI participants’ language subtest scores fell within the low end of the average range, with few (a quarter of the dysarthric group) being classified as having a language disorder per se. Mild language deficits more than 2 years after severe TBI have been reported previously (Ewing-Cobbs & Barnes, 2002; Sullivan & Riccio, 2010 for reviews), including in the domains of receptive and expressive vocabulary (Catroppa & Anderson, 2004; Ewing-Cobbs & Barnes, 2002), with little recovery in those with severe injuries (Anderson et al., 2004). Our findings therefore confirm that language problems can persist longterm after moderate to severe TBI.

### 4.2 Sentence generation and tractography findings

The most impaired domain was sentence generation (Formulated Sentences subtest), with a third of participants with dysarthria showing significant difficulties. This subtest requires the combination of short term auditory-verbal memory, syntactic and semantic skills, as well as retrieval skills to be able to formulate a correct sentence. These demands are added to the constraints of being given a particular word or phrase to use in the sentence and picture stimuli providing a scene or context for the participant’s response. Impairment on this subtest can therefore reflect difficulties in any or several of these skills. The deficits may also be related to this population’s reported long term difficulties forming narratives (Dennis & Barnes, 2000; Ewing-Cobbs et al., 1998; Levin et al., 2001), but only further testing will enable us to confirm this hypothesis.

It is noteworthy that the relationship between dysarthria and expressive language impairment does not however appear to be causal in this sample. Examination of errors revealed that these participants scored poorly on this subtest due to grammatical and semantic errors rather than articulation difficulties or fatigue due to their dysarthria. These observations and our tractography data suggest that damage to the motor speech networks – corticobulbar and corticospinal tracts – can be concomitant with damage to other language networks involved in initiation, word retrieval, and semantic knowledge. If confirmed in a larger sample, these results indicate that a significant subset of dysarthric children post TBI are at an increased risk for reduced language function, particularly impacting on sentence production.

In our sample, volume reduction in both the arcuate fasciculus and the corpus callosum predicted best performance on the Formulated Sentences subtest, with each predictor making a significant and unique contribution. This suggests that there is a cumulative effect of combined damage, which results in the worst outcome. One possible interpretation of these findings is that corpus callosum damage prevents reorganisation and use of the right hemisphere tract to compensate for the left-sided damage. Another is that both intra- and inter-hemispheric white matter pathways equally contribute to sentence generation.

In healthy adults, increased left lateralization of the AF is associated with poorer performance on a word list recall test (Catani et al., 2007). In healthy children, diffusion metrics of the left arcuate correlate with phonological awareness skills (Yeatman et al., 2011). In contrast, the relationship between arcuate diffusion metrics and sentence production has, to our knowledge, not been reported in healthy adults or children. Some studies have reported a relationship between left arcuate damage and aphasia in adults post-stroke (Kim et al., 2011). For instance, syntactic deficits in adults with primary progressive aphasia has been attributed to damage to the superior longitudinal fasciculus (Wilson, Galantucci, Tartaglia, & Gorno-Tempini, 2012). In our participants with TBI, early damage to the left arcuate could have impacted semantic knowledge, syntactic processing, or both.

### 4.3 Semantic knowledge and tractography findings

Sustaining a TBI with subsequent dysarthria also affected the domain of semantics, measured here using the Word Classes subtest. These results are consistent with those of another study of participants with TBI sustained in childhood that examined outcomes on a semantic similarities subtest (Catroppa & Anderson, 2004). Children with moderate and severe injuries scored in the lower end of the normal range on average at 2 years post-injury. We cannot ignore, however, that the Word Classes subtest used here also requires working memory to “hold” the target words whilst selecting the semantically related items. It was therefore difficult to disentangle the purely semantic vs. memory errors for participants in our post TBI sample. Working memory is commonly affected after childhood TBI (e.g. Mandalis, Kinsella, Ong, & Anderson, 2007; Moran & Gillon, 2004) and its contribution to language is frequently discussed in children with developmental language disorders (e.g., Lum, Conti-Ramsden, Page, & Ullman, 2012). The relative deficits in word comprehension observed in our dysarthric group could therefore be due to a combination of factors.

The significant correlation between volume of the left uncinate fasciculus and performance on the Word Classes subtest suggests that damage to this tract may result in semantic impairment, consistent with distributed models of semantic knowledge implicating the “ventral route” (Saur et al., 2008). The strong association between right arcuate FA and scores on this subtest was, in contrast, unexpected based on current neuroanatomical models of adult language processing. One hypothesis is that reduced FA in the right arcuate post TBI is related to lower verbal memory, as in healthy adults (Catani et al., 2007), and that verbal memory reduction significantly compromises performance on the Word Classes subtest. Similarly, a positive correlation has previously been reported between FA of the SLF bilaterally and the Word Classes subtest in individuals with specific language impairment (SLI, Verhoeven et al., 2011). In our study, the verbal memory (arcuate related) deficits therefore seem to predict performance as well as semantic (uncinate related) deficits. Here again, children with dysarthria were significantly more impaired than their non-dysarthric peers, a finding that we could attribute to more widespread damage to the white matter across both ventral and dorsal language pathways as well as motor speech pathways.

### 4.4 Recalling Sentences and tractography findings

The ability to repeat sentences verbatim was not an area of concern for the TBI group here, consistent with other studies reporting that sentence repetition was relatively spared in children whose TBI occurred after the preschool period (Ewing-Cobbs & Barnes, 2002; Sullivan & Riccio, 2010) – as in our sample.

The positive correlation between FA in the right AF and scores on the Recalling Sentences subtest is consistent with the positive correlation reported between having symmetrical arcuate fasciculi and performance on a word list recall test in healthy adults (Catani et al., 2007). It is possible that after TBI, the right arcuate fasciculus compensates for the verbal memory function of the damaged left arcuate, resulting in no deficits irrespective of the presence of dysarthria. Overall, the diffusion changes in the right arcuate fasciculus in relation to language (Recalling Sentences and Word Classes subtests) were unexpected. Our current interpretation is that two intact arcuates are related to better performance than only one intact arcuate – whether left or right.

### 4.5 Effect of age and age at injury on language outcome

In our sample, age at injury ranged from 3 to 16 years, and injury severity was mainly moderate–severe. Nevertheless, we found little evidence of a relationship between either of these variables and language outcome. Anderson et al. (2011) have suggested that for children with non-focal injury (such as TBI), younger children may be more at risk of deficits than those with later injuries. It has also been suggested that skills that are developing or not developed yet when TBI occurs are the most vulnerable (Ewing-Cobbs & Barnes, 2002). Here one significant correlation was found between age at injury and volume of the left arcuate fasciculus, possibly reflecting a more immature tract at the time of TBI. If confirmed in other white matter pathways, arrested development could explain the early vulnerability described for other cognitive functions (Anderson et al., 2012).

### 4.6 Corpus callosum and language functions

It is widely known that TBI affects the integrity of the corpus callosum (Wu et al., 2011), although the relationship between damage to this tract and language is rarely examined. More recently, our group has reported a relationship between CELF-IV total scores in adolescents born preterm and corpus callosum integrity (Northam et al., 2012), suggesting that abnormal development of this structure early in life can have a negative impact on language development overall. Altogether our data, and that of others (e.g., Hinkley et al., 2012; Josse, Seghier, Kherif, & Price, 2008) suggest that although not traditionally a language-related tract, the corpus callosum does play an important role in connectivity between left and right hemisphere language regions, and therefore should not be dismissed when attempting to investigate neural predictors of language outcome in children with brain injury.

### 4.7 Uncinate fasciculus and language functions post TBI

Despite evidence of bilateral changes in the left and right uncinate in participants with dysarthria relative to the healthy control group, consistent with diffuse axonal injury (Seo et al., 2012), we had little evidence that these changes were related to language outcome as measured here. A preoperative study in 13 adults with tumour has questioned whether the uncinate is a tract “essential” for language function (Duffau, Gatignol, Moritz-Gasser, & Mandonnet, 2009). Direct stimulation of the tract did not elicit language disturbance, and complete resection of the tract did not result in postoperative language deficits. In addition, it has been suggested that this tract is crucial to the retrieval of proper names rather than conceptual knowledge (Papagno et al., 2011). Furthermore, a recent study reported that diffusion changes to the ventral pathway (including the uncinate fasciculus and the extreme capsule system) were not related to language impairments in adolescents born preterm (Northam et al., 2012). It therefore seems that although this tract may be involved in a wide semantic network in adults, perhaps other networks can compensate for its role after an injury during development.

### 4.8 Specificity of findings

Our findings do not allow us to conclude that either the language deficits or the track changes are specific. The widespread nature of injuries after TBI (e.g. see VBM findings in Morgan et al. (2013)) is well known. A significant proportion of the variance in language outcome may therefore be explained by additional changes in tracks not examined here. However, without any a priori hypothesis, measuring changes across the whole brain increases the problem of multiple comparisons, and we therefore chose to focus on well-known language-related tracts. Of note, we failed to find an association between language scores and (i) severity of injury and (ii) global white matter volume, suggesting that language deficits here are not merely a consequence of more severe injury.

One could question whether reduced language performance could be a consequence of other cognitive deficits not measured in this study. Here we can only argue that language problems are beyond those seen in IQ. Further studies examining attention, executive function, and working memory for instance would be needed to fully account for the presence of co-morbid disorders post TBI. Examining the integrity of other tracks in future studies may help refine the brain markers of language impairment after childhood brain injury.

With regards to the relationship between speech and language, we identified associations between measures from language (arcuate) and motor tracts (speech/face-related). This relationship may be explained by the fact that the AF and the CST/CBT are anatomically close at the level of the corona radiata. Examination of these tracts at different axial levels may help clarify why children with dysarthria are more at risk of reduced language scores than those without.

### 4.9 Study limitations

This study was the first, to the author’s knowledge, to investigate the impact of pediatric TBI on the arcuate fasciculus and its relationship to language outcomes. A restriction of the study was the selected nature and limited size of the recruitment sample, given the effort to match the groups with and without dysarthria. Replication of findings in a larger sample size is warranted to confirm the present results. It is also important to be aware of the inherent limitations of tractography. This method is an indirect measure of underlying white matter damage and is unable to delineate between underlying axonal and myelination damage (Beaulieu, 2002).

Finally, it is important to note that track volume measured here is an indirect measure of macroscopic change, and its biological interpretation is controversial and remains speculative at this stage (Jones, Knösche, & Turner, 2012). Here we cannot rule out that volume reductions are a consequence of grey matter dysfunction or lesion, rather than Wallerian degeneration or tract damage.

### 4.10 Clinical implications: double blow to the corpus callosum and arcuate post TBI

Participants with dysarthria were at greater risk of poor language outcomes than those without, mainly in the area of sentence production. Overall, it seems that individuals with damage to the left arcuate fasciculus and corpus callosum may be less likely to show spontaneous functional reorganisation. They may benefit from earlier screening, detection and management of language impairments during the acute phase in order to optimise longer-term functional outcomes.

### 4.11 Conclusions

Co-morbid speech motor and language deficits after TBI appeared to be a result of widespread damage affecting both motor (corticobulbar and corticospinal) and language tracts in this group. Given that 2/3 of the sample with dysarthria had no clinically defined language impairment, we have little evidence to support that chronic speech motor difficulties per se systematically affect language development. For the remaining third, early prognostic biomarkers may include corpus callosum and arcuate volume reduction, and early language intervention may be warranted.

## Figures and Tables

**Table 1:** Demographic and injury characteristics for each group.

	Control	TBI+	TBI−
*Sex*			
Male	8	8	6
Female	9	9	9

*Age at scanning*			
Mean	18;4 years	17;10 years	18;9 years
SD	4;10 years	4;1 years	4;4 years

*Age at TBI*			
Mean	–	9;4 years	10;11 years
SD	–	4;4 years	3;11 years
Range		3–16 years	4–16 years

*Severity of injury*			
Mild	–	1	1
Moderate–severe	–	16	14

*Type of TBI*			
Fall	–	6	3
MVA pedestrian	–	6	6
MVA passenger	–	3	3
Other	–	2	3

*Abbreviations*: SD = standard deviation; MVA = Motor Vehicle Accident; TBI+ = traumatic brain injury with dysarthria; TBI− = traumatic brain injury without dysarthria. Age in years; months.

**Table 2:** Mean language and IQ scores for the three groups.

Measure	Control	TBI+	TBI−
*CELF standard score*			
Mean	104.9	88.6	96.5
SD	11.00	12.2	11.7
95% CI	99.3–110.6	82.3–94.9	90.1–103.0

*WASI standard score*			
Mean	103.7	90.7	99.6
SD	13.2	12.0	12.7
95% CI	96.9–110.5	84.5–96.8	92.6–106.6

TBI+ = traumatic brain injury with dysarthria, TBI− = traumatic brain injury without dysarthria. CI, confidence interval.

**Table 3:** Group comparisons of tractography-derived values.

	Main effect of group^a^	Post hoc comparisons^b^		
		TBI+ vs. controls	TBI+ vs. TBI−	TBI− vs. controls
*Left uncinate fasciculus*				
FA	*F* = 6.10, *p* = 0.005	*p* = 0.003	ns	ns
Volume	*F* = 0.11, ns	ns	ns	ns

*Right uncinate fasciculus*				
FA	*F* = 0.01, ns	ns	ns	ns
Volume	*F* = 8.71, *p* = 0.001	*p* < 0.001	ns	ns

*Left arcuate fasciculus*				
FA	*F* = 4.28, *p* = 0.02	ns	*p* = 0.03	ns
Volume	*F* = 6.00, *p* = 0.005	*p* = 0.005	*p* = 0.06	ns

*Right arcuate fasciculus*				
FA	*F* = 4.78, *p* = 0.01	*p* = 0.01	ns	ns
Volume	*F* = 0.51, ns	ns	ns	ns

*Corpus callosum*				
FA	*F* = 1.40, ns	ns	ns	ns
Volume	*F* = 6.79, *p* = 0.003	*p* = 0.002	*p* = 0.055	ns

a ANOVAs for FA comparisons; ANCOVAs (with global white matter as a covariate) for volume comparisons.

b Bonferroni corrected; ns, not significant (*p* > 0.10).

## Appendix A Supplementary material

Supplementary Table 1 Summary of individual imaging reports from each TBI group. MRI was used unless specified.

Supplementary Fig. 1 Boxplots of language subtest standardised scores across the three groups. Lines indicate median, box interval indicates range between 25th and 75th percentile. Outliers are illustrated by circles and asterisks.

Supplementary Fig. 2 Relationship between IQ and language scores in each group.

Supplementary Fig. 3 Boxplots for volume (top) and mean fractional anisotropy (FA) across the three groups. Lines indicate median, box interval indicates range between 25th and 75th percentile. Outliers are illustrated by circles and asterisks.

Supplementary Fig. 4 Relationship between tractography metrics and language scores (A, Formulated Sentences; B, Word Classes; C, Recalling Sentences). Only significant correlations are shown (see text for coefficients).
