# Supplementary material for: Pediatric traumatic brain injury: Language outcomes and their relationship to the arcuate fasciculus
Source: Brain Lang. 2013 Dec;127(3):388–98. doi: 10.1016/j.bandl.2013.05.003 (PMC3988975; doi:10.1016/j.bandl.2013.05.003)
Supplement: Supplementary Table 1 — Summary of individual imaging reports from each TBI group. MRI was used unless specified. [file mmc1.docx]

**Supplementary Table 1**. Summary of individual imaging reports from each TBI group. MRI was used unless specified.

| **TBI group without dysarthria** | |
| --- | --- |
| 1 | - Extensive bifrontal haemorrhagic contusion extending posteriorly into genu of corpus callosum and R cingulate gyrus. - Signal abnormality hypothalamus; R caudate; R thalamus. |
| 2 | Multiple lesions consistent with intra axial petechial haemorrhage in:   - Posterolateral aspect of L temporal lobe. - Anterosuperior aspect of L frontal lobe. - Inferior aspect of R frontal lobe. |
| 3 | - Inferior R frontal fracture with underlying contusion and small amount vasogenic oedema. |
| 4 | - Fracture and associated fluid through lateral aspect of R maxillary sinus. |
| 5 | (CT):   - Avulsion fracture of medial portion of L occipital condyle. - Small volume of subarachnoid in extra axial spaces overlying L frontoparietal regions. |
| 6 | - No focal lesion is identified, normal study. |
| 7 | (CT):   - Superficial haematoma in L temporal area. - L subdural approximately 6.3cm long and 1.2cm deep. |
| 8 | (CT):   - Multiple skull fractures. - Bifrontal and L temporal haemorrhagic contusions. - L extra axial haemorrhage and generalised swelling. |
| 9 | - R parietotemporal fracture involving roof of orbit and features suggesting possible parenchymal oedema involving posterior fossa more than supratentorial parenchyma. |
| 10 | - No abnormality identified. - No intracranial lesion is seen. - No parenchymal or extra axial haemorrhage. |
| 11 | (CT):   - Acute R dural haematoma with mass affect exerted in L frontal lobe. - Mild line shift, effacement of L lateral ventricle and some loss of grey white differentiation in L frontal lobe owing to mass effect from extradural haematoma. - Soft tissue swelling of haematoma in R superior parietal region but no underlying intracranial abnormality. |
| 12 | (CT):   - Small amount of high density material adjacent to anterior aspect of L cerebellar hemisphere extending into region of L sigmoid sinus (possibly a subdural collection or less likely small amount of subarachnoid blood). - High density area R frontal lobe level with frontal horns (possible further small area of subdural blood). - Stellate involving L orbital roof with a linear extension within L parietal bone superiorly towards the vertex. - Medially fracture lines extend through L sided lamina papyracea into region of ethmoid air cells. - Inferiorly the fracture line extends through lateral wall of orbit as well as lateral wall of the L maxillary antrum. - Further fractures are identified involving anterior and lateral wall of R maxillary antrum and both nasal bones. |
| 13 | (CT)   - No intracranial abnormalities or fractures. |
| 14 | (CT):   - No focal lesions identified. - No intra or extra axial bleed. No fractures. - Normal brain study. |
| 15 | (CT):   - Suspected fracture through L petrous temporal bones with fluid/soft tissue material noted within L mastoid air cells and middle ear cleft. - Fracture line extending from L mastoid air cells vertically involving L posterior temporal and parietal regions. - Localised underlying extra-axial haematoma measuring approximately 9mm in width and associated with small locule of intracranial air. - Generalised cerebral oedema. - Posterior fossa contents appear swollen, although fourth ventricle still visualised to be patent. - Cerebellar tonsils may be slightly low-lying. - Good visualisation of suprasellar cistern but rest of the peri-mesencephalic cisterns are almost completely effaced due to cerebral oedema. - Cerebral parenchyma is generally of low density and there is some loss of grey/white differentiation in the temporal lobes bilaterally. - Generalised effacement of the sulcal and gyral pattern in keeping with cerebral swelling. - Third ventricle is slit-like and both lateral ventricles are also rather attenuated. - Minimal asymmetry of lateral ventricles due to slightly increased effacement of L lateral ventricle, presumably related to L extra-axial collection. - 2-3mm midline shift to R. - Incidental pineal and choroid plexus calcification noted. |
| **TBI group with dysarthria** | |
| 1 | - Multiple skull vault fractures: L frontal and parietal bones + R parietal bones identified. - Small pneumocephalus detected adjacent to fracture site. - Small L subdural haemorrhage at L anterior parietal area with multiple scattered haemorrhagic contusions in both cerebral hemispheres, particularly on R posterior temporal lobe. - Minimal subarachnoid haemorrhage. - R and L parietal scalp swelling. - Generalised cerebral oedema. - Suggested presence of haematoma/clot. |
| 2 | - Some soft tissue swelling in scalp overlying L temporal squamous region. - 4th ventricle is central in posterior fossa. - Spacious appearance in basal cistern region noted. - Slight loss of grey/white interface above the tentorium, with suggestion of mild cerebral oedema. |
| 3 | (CT):   - Small subdural collection on L side of anterior cranial fossa is now iso-intense to parenchyma, and cannot be clearly defined. - Three small locules of air extra-cranially. - Facial fractures. |
| 4 | (CT):   - Small haemorrhagic contusions in high R frontal region and R cerebellar hemisphere. - Subdural haemorrhage, mainly in posterior interhemispheric fissure and along tentorium. |
| 5 | - Soft tissue swelling overlying frontal bones extending into R pre-septal peri-orbital soft tissue. - No skull fracture of the adjacent cranial vault or elsewhere. - Small R frontal deep white matter petechial haemorrhage. - No evidence of midline shift, mass effect or extra-axial collections. |
| 6 | (CT):   - Extensive base of skull fractures extending through the L poster lateral orbit. - Involvement of optic canals. Possible hematoma apex of R orbit. - Intracranial subarachnoid and possible subdural blood. - R ventriculostomy. |
| 7 | (CT):   - Small focus of haemorrhage in splenium of corpus callosum. - Gyriform contusion on R parietal lobe towards vertex. Few areas of linear density in L parietal lobe towards vertex and difficult to distinguish if parenchymal contusions or due to subarachnoid bleed. - Increased density to R of interhemispheric fissure towards vertex, suggestive of small subdural bleed. - Overall, shearing type of intracranial haemorrhages. |
| 8 | (CT):   - Multiple haemorrhagic contusions in cerebral hemispheres. - Midline focus of haemorrhage that may lie in aqueduct of Sylvius or in peri-sylvian region. |
| 9 | (CT):   - R globus pallidus haemorrhage. |
| 10 | (CT):   - Extensive compound basal skull fractures extending to fault at least on L. - No intracranial haematoma. - Some evidence suggesting brain swelling. |
| 11 | (CT):   - Extensive soft tissue swelling over R side of cranium. - Depressed fracture in R frontal region. - Multiple areas of contusion are seen in R frontal lobe. - Some loss of grey/white differentiation suggesting oedema. - Subarachnoid blood in temporal horn in R lateral ventricle and also in region of interpeduncular cistern. |
| 12 | (CT):   - Intraventricular haemorrhage with normal positioning of a transcranial shunt. |
| 13 | (CT):   - Several areas of punctate haemorrhages in cerebellar and cerebral hemispheres: - Right cerebellar hemisphere white matter; possibly involving L pons. - Right inferior frontal lobe, and R thalamic region, adjacent to posterior limb of the internal capsule. - Smaller foci suspected in L internal capsule region. - Small amount of subarachnoid blood in interpeduncular cistern. Scalp swelling over R parietotemporal region. - Findings suggestive of diffuse axonal injury. |
| 14 | - Moderately large frontal extradural haematoma resulting in significant mass effect with bowing of midline to L, effacement of cortical sulci and loss of peri-mesencephalic cisterns. - Several scattered parenchymal contusions in keeping with diffuse axonal injury. - Undisplaced R frontal bone fracture and R intraorbital gas likely secondary to fracture of medial wall of the R orbit. This is associated with R proptosis and R peri-orbital haematoma. |
| 15 | (CT):   - R scalp bruising. - No abnormality detected intracranially. - Note: lack of aeration in R mastoids. - No focal lesion but some diffuse cerebral swelling. |
| 16 | - Multiple small haemorrhage foci in cerebellar hemispheres bilaterally. - Lesions in R thalamus and in R frontal lobe at the grey/white junction (some appear to have slight associated oedema or local swelling, and overall have appearance of small contusions). |
| 17 | - Extensive damage to brain. - Regions of abnormal signal at grey white interface within brain stem and corpus callosum are consistent with diffuse shearing injury. |

Abbreviations: CT, Computed Tomography. L, left; R, right.
