# Supplementary material for: Pediatric traumatic brain injury: Language outcomes and their relationship to the arcuate fasciculus
Source: Brain Lang. 2013 Dec;127(3):388–98. doi: 10.1016/j.bandl.2013.05.003 (PMC3988975; doi:10.1016/j.bandl.2013.05.003)
Supplement: Supplementary Fig. 1 — Boxplots of language subtest standardised scores across the three groups. Lines indicate median, box interval indicates range between 25th and 75th percentile. Outliers are illustrated by circles and asterisks. [file mmc2.docx]

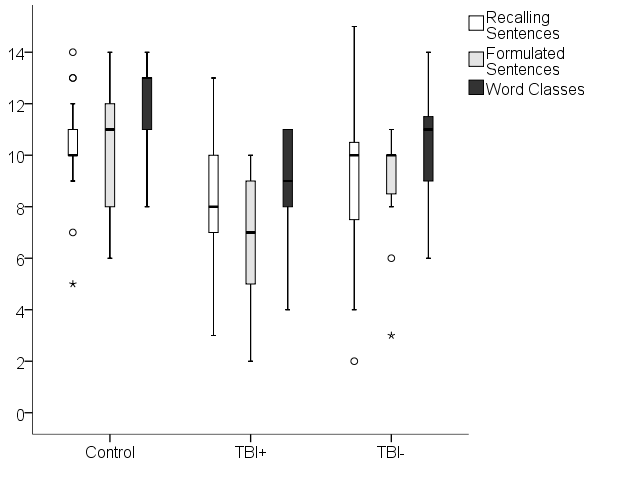


**Supplementary Figure 1.** Boxplots of language subtest standardised scores across the three groups. Lines indicate median, box interval indicates range between 25^th^ and 75^th^ percentile. Outliers are illustrated by circles and asterisks.
