# Supplementary figures and images for: Pediatric traumatic brain injury: Language outcomes and their relationship to the arcuate fasciculus
Source: Brain Lang. 2013 Dec;127(3):388–98. doi: 10.1016/j.bandl.2013.05.003 (PMC3988975; doi:10.1016/j.bandl.2013.05.003)

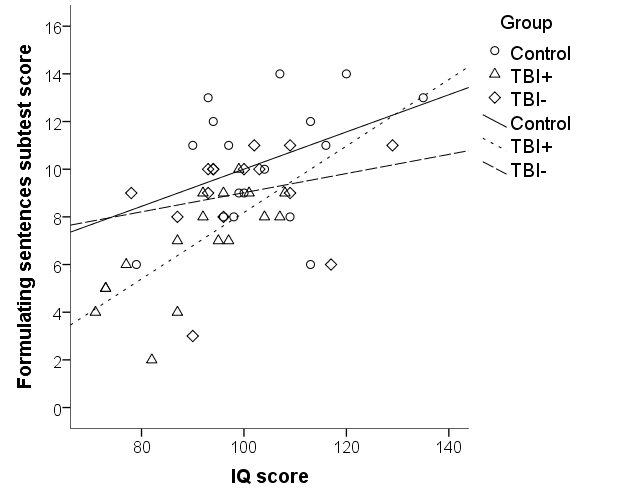

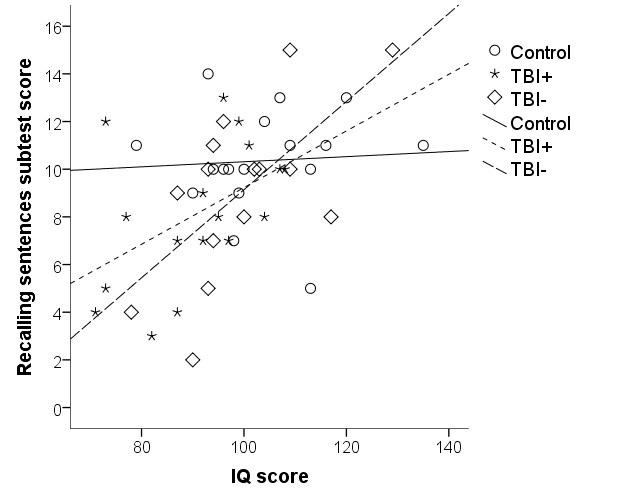

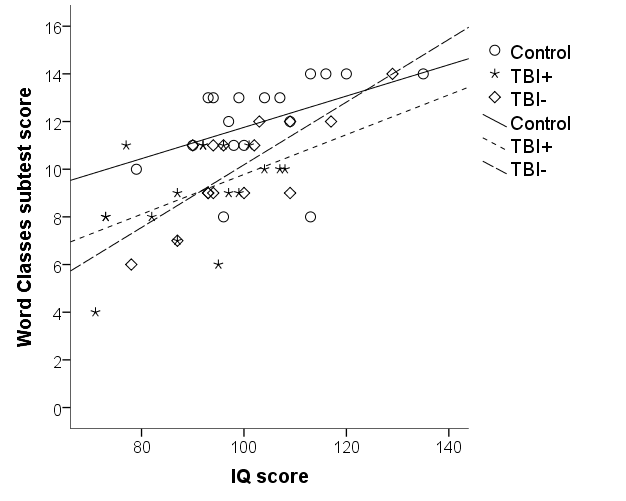


**Supplementary Figure 2.** Relationship between IQ and language scores in each group.

Supplement: Supplementary Fig. 2 — Relationship between IQ and language scores in each group. [file mmc3.docx]
