# Supplementary material for: Pediatric traumatic brain injury: Language outcomes and their relationship to the arcuate fasciculus
Source: Brain Lang. 2013 Dec;127(3):388–98. doi: 10.1016/j.bandl.2013.05.003 (PMC3988975; doi:10.1016/j.bandl.2013.05.003)

**Supplementary Figure 3.** Boxplots for volume (top) and mean Fractional Anisotropy (FA) across the three groups. Lines indicate median, box interval indicates range between 25^th^ and 75^th^ percentile. Outliers are illustrated by circles and asterisks.

Corpus callosum Language tracks


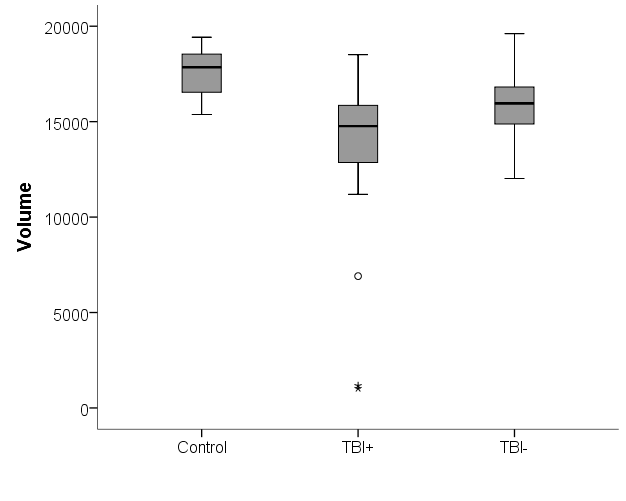

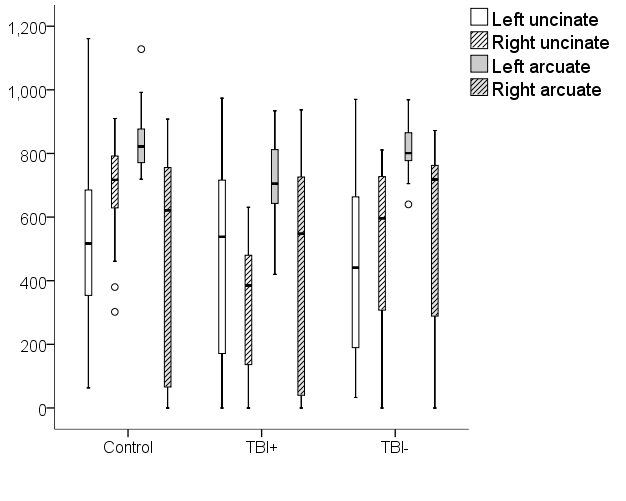

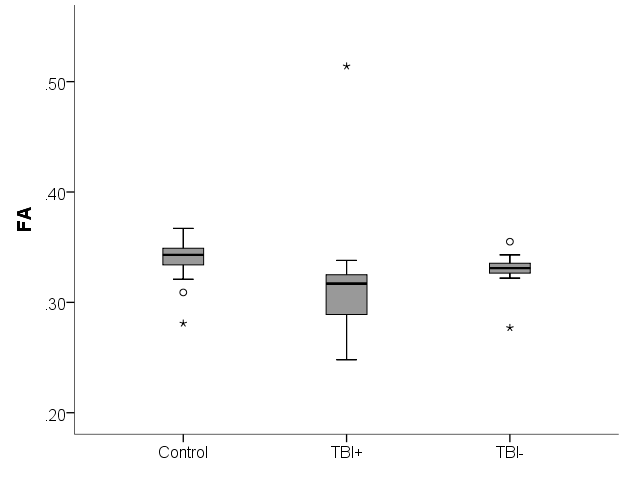

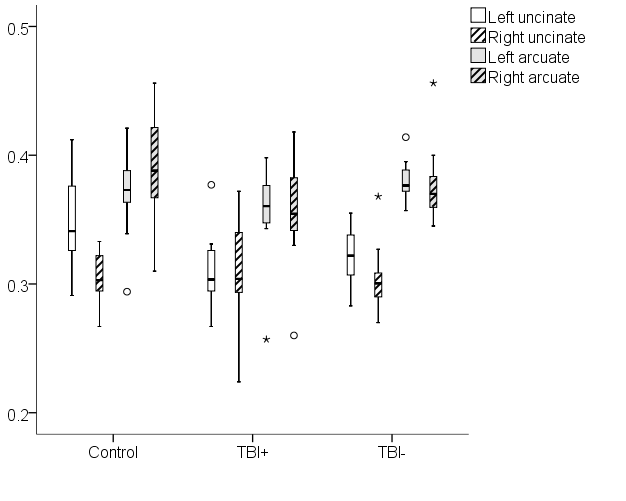

Supplement: Supplementary Fig. 3 — Boxplots for volume (top) and mean fractional anisotropy (FA) across the three groups. Lines indicate median, box interval indicates range between 25th and 75th percentile. Outliers are illustrated by circles and asterisks. [file mmc4.docx]
