# Supplementary material for: Pediatric traumatic brain injury: Language outcomes and their relationship to the arcuate fasciculus
Source: Brain Lang. 2013 Dec;127(3):388–98. doi: 10.1016/j.bandl.2013.05.003 (PMC3988975; doi:10.1016/j.bandl.2013.05.003)
Supplement: Supplementary Fig. 4 — Relationship between tractography metrics and language scores (A, Formulated Sentences; B, Word Classes; C, Recalling Sentences). Only significant correlations are shown (see text for coefficients). [file mmc5.docx]

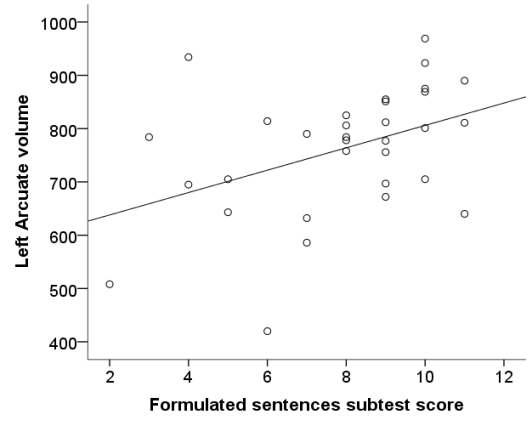

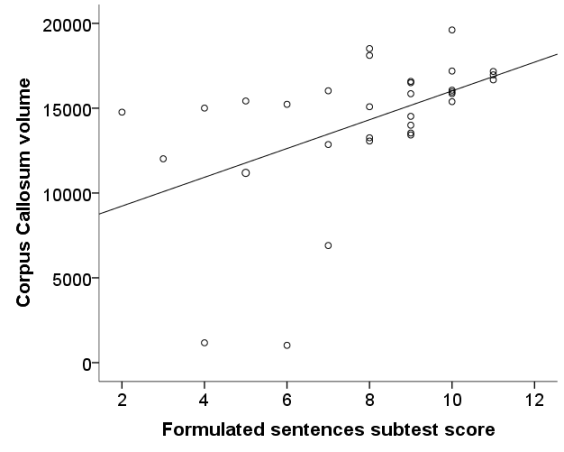


A

B


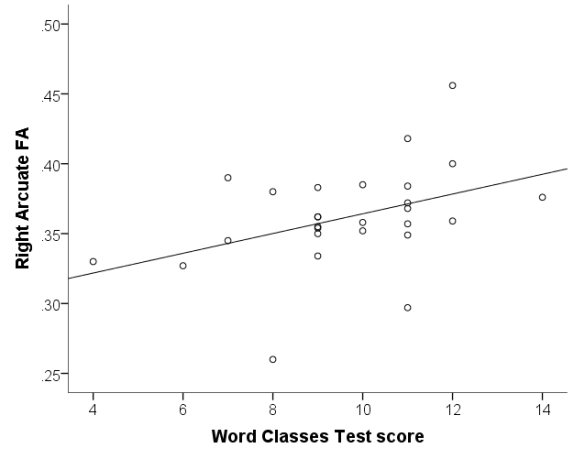

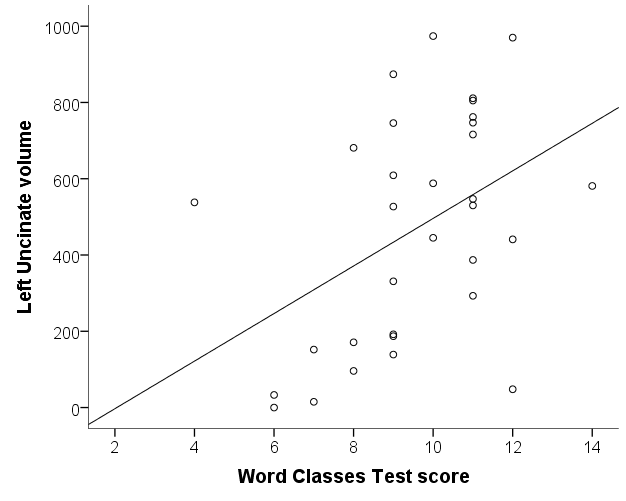

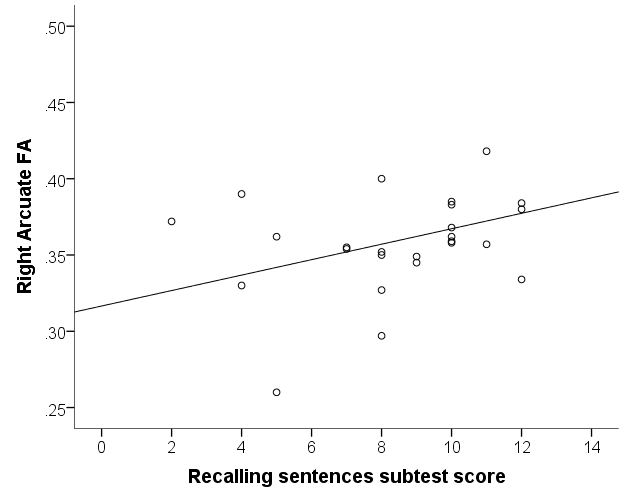


C

**Supplementary Figure 4.** Relationship between tractography metrics and language scores (A, Formulated Sentences; B, Word Classes; C, Recalling Sentences). Only significant correlations are shown (see text for coefficients).
